# Is the fusion order of the cranial and caudal levels different in two-level anterior cervical discectomy and fusion for cervical spondylopathy? A retrospective study

**DOI:** 10.1186/s13018-021-02657-2

**Published:** 2021-08-16

**Authors:** Xia-Qing Sheng, Yang Meng, Hao Liu, Bei-Yu Wang, Yi Yang, Xin Rong, Ying Hong

**Affiliations:** 1 Department of Orthopedic Surgery, West China Hospital, Sichuan University, No. 37 Guo Xue Xiang, Chengdu, 610041 Sichuan China; 2 West China School of Nursing, Sichuan University, No. 37 Guo Xue Xiang, Chengdu, 610041 Sichuan China; 3 Department of Anesthesia and Operation Center, West China Hospital, Sichuan University, No. 37 Guo Xue Xiang, Chengdu, 610041 Sichuan China

**Keywords:** Cervical, Anterior cervical discectomy and fusion, Fusion order, Zero-profile device, Segment slope, Cranial and caudal levels

## Abstract

**Study design:**

Retrospective study.

**Objective:**

This study aimed to compare the fusion order between the cranial and caudal levels in two-level anterior cervical discectomy and fusion (ACDF) with a zero-profile device in the treatment of cervical spondylopathy.

**Summary of background data:**

Fusion is the standard used to judge the success of ACDF. However, the fusion order in two-level ACDF remains uncertain. The mechanical environment of different levels is different, which may affect the fusion rate or fusion order.

**Methods:**

From 2014 to January 2019, data of consecutive patients with two-level cervical disk degenerative disease who underwent ACDF were retrospectively reviewed. Radiological assessments were based on the range of motion of the fusion level, segment slope, and disk height, and complications were assessed. Data were analyzed using the paired *t*, Mann-Whitney *U*, *χ*^2^, Fisher exact, and rank-sum tests and logistic regression analysis.

**Results:**

In total, 118 patients were ultimately enrolled for analysis in the study. The respective fusion rates of the cranial and caudal levels were 26.27% and 10.17% (*p* < 0.05) at 3 months, 58.47% and 42.37% (*p* < 0.05) at 6 months, 86.44% and 82.20% (*1* 0.05) at 1 year, and 92.37% and 89.83% (*p* > 0.05) at the last follow-up. Multivariate logistic regression analysis indicated that the preoperative segmental slope and cranial level were independent risk factors for non-fusion. The adjacent segment degeneration (ASD) and subsidence rates were comparable between the two levels.

**Conclusion:**

The caudal level had a slower fusion process than the cranial level. A higher preoperative segment slope was a risk factor for fusion. However, the subsidence and ASD rate were comparable between the caudal and cranial levels in the two-level ACDF.

## Introduction

Anterior cervical discectomy and fusion (ACDF) is a classic procedure for treating cervical spondylosis with good clinical outcomes and improves patient’s quality of life [1–5]. The success of ACDF relies on the achievement of arthrodesis. Although pseudarthrosis can often be asymptomatic, it may finally lead to mechanical pain, poor patient satisfaction, and implant failure requiring revision [6–8]. In the presence of these clinical problems, it is imperative to go back to basic research, analyze the causes of the problems, and propose solutions so as to guide clinical practice again, which is in line with the model of translational medicine [9].

In previous studies, the fusion rate of two-level ACDF varied; a systematic review [10] reported a mean fusion rate of 90.1%, which ranged from 30 to 100%. Smoking, osteoporosis, diabetes, preoperative range of motion (ROM), and preoperative T1 slope are the factors that influence the fusion rate or fusion speed [11–16].

In addition, different spine levels have unequal mechanical environments, which may lead to a discrepancy in the fusion process among levels. A few studies have found that pseudarthroses often appear at the caudal level in multi-level ACDF [17, 18]. However, the fusion order in two-level ACDF is rarely known. Therefore, this study aimed to explore the fusion rate and fusion order between the cranial and caudal levels in two-level ACDF for cervical spondylosis. The hypothesis was that the caudal level has a slower fusion process than the cranial level, according to which an improved strategy of bone graft could be made.

## Materials and methods

### Study design and patient population

This retrospective study was conducted from January 2014 to January 2019. The study protocol was approved by the Ethics Committee on Biomedical Research of the West China Hospital of Sichuan University, and informed consent was obtained from the patients. In total, 129 consecutive inpatients from a single center had undergone two-level ACDF with failed conservative treatment for at least 6 weeks. Surgical indications were symptomatic radiculopathy and/or myelopathy caused by contiguous two-level cervical degenerative disk disease between C2 and C7 based on clinical performance, preoperative radiography, computed tomography (CT), and magnetic resonance imaging (MRI) findings. The exclusion criteria were (1) a history of cervical spine surgery and (2) other cervical diseases including infection, cervical spinal tumor, cervical fracture, or other severe systematic diseases.

### Surgical techniques

In our hospital, all procedures were performed by a single surgeon with a standard right-side anterior approach after general anesthesia. A transverse incision was made along the skin on the right and front of the patient’s neck. Complete discectomy was conducted at the indicated levels by removing the disk tissue, posterior longitudinal ligament, and osteophytes to achieve thorough decompression. A high-speed burr was used to prepare the endplates, and a properly sized Zero-P implant (Synthes, Oberdorf, Switzerland) filled with a composite synthetic bone graft (beta-tricalcium phosphate, ChronOS; DePuy Synthes, Paoli, CA, USA) was inserted into the indicated levels. Proper placement of the implants was verified by C-arm fluoroscopy. Finally, the muscle was closed with sutures, and the subcutaneous tissue and skin were then sutured layer by layer after drainage insertion. Recombinant human bone morphogenetic protein-2 (rhBMP-2) was not used concurrently. All patients wore neck braces for 3 months.

### Radiological parameters

**Fig. 1:**
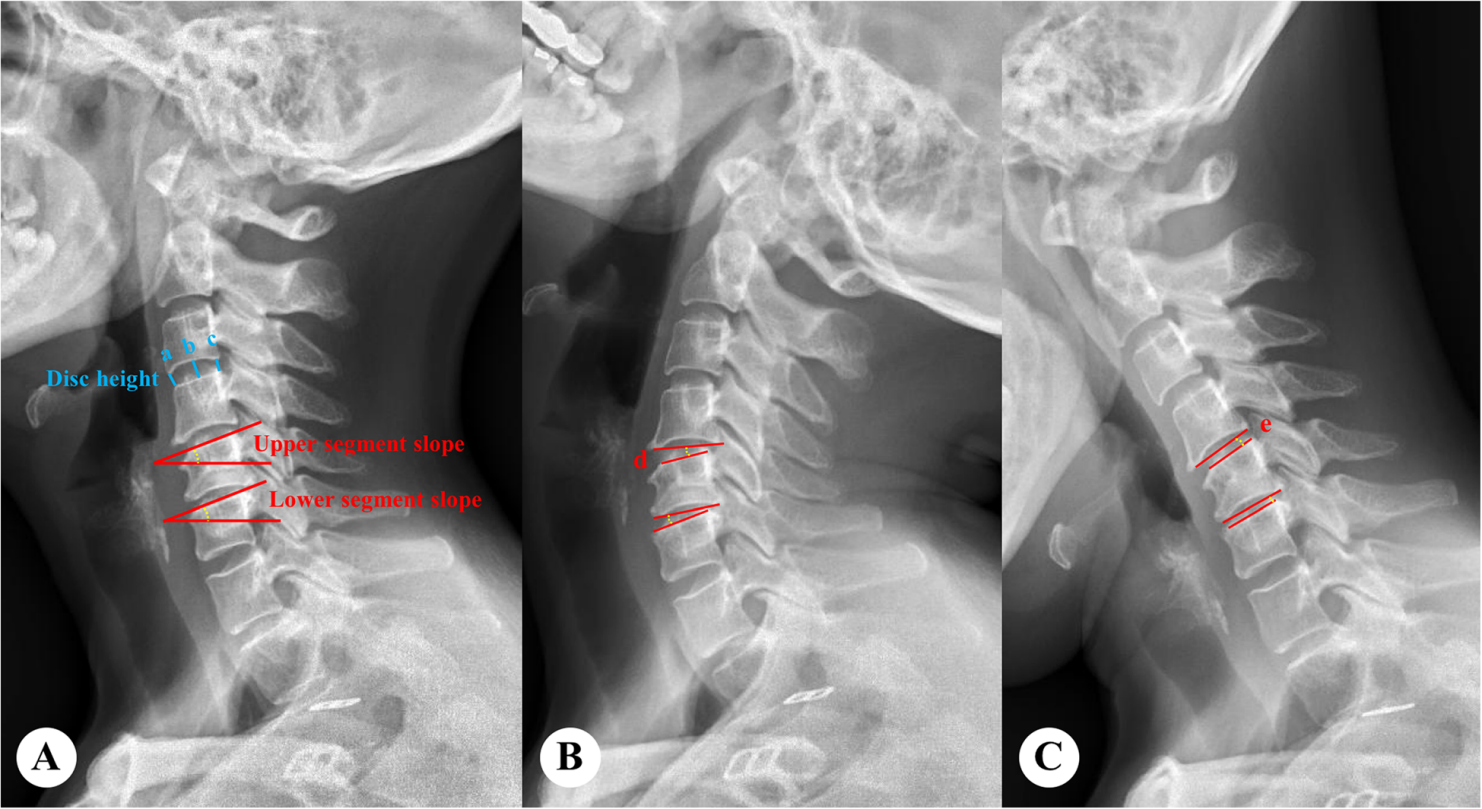
**A** Disk height is calculated as (a+b+c)/3. The segment slope is defined as the angle between the horizontal line and the extension line of the upper endplate of the middle (or caudal) vertebral body. **B**, **C** Range of motion is calculated as d–e

Radiographical outcomes were measured before surgery; at 1 week, 3 months, 6 months, and 1 year after surgery; and at the last follow-up. Radiological parameters included disk height (DH), ROM of the fusion level, and segment slope. Adjacent segment degeneration (ASD) was defined as a progressive or new-onset posterior osteophyte, decrease in DH > 25%, disk herniation, disk signal change, or anterior/posterior longitudinal ligament calcification at the final follow-up compared with the preoperative state on lateral radiographs or MRI scans [19]. Fusion was defined as ROM of the fusion level < 2° on lateral flexion/extension radiographs and the presence of trabecular bridging on radiographs or CT scans [10, 20]. Subsidence was defined as DH loss > 2 mm from 1 week to the last follow-up postoperatively. The segment slope was defined as the angle between the horizontal line and the extension line of the upper endplate of the middle (or caudal) vertebral body (Fig. 1). PACS version 4.0 (GE Healthcare, Milwaukee, WI, USA) was used to measure the radiological parameters.

### Statistical analysis

The results are presented as the mean ± standard deviation. A paired *t* test was used to analyze normally distributed contiguous data between the cranial and caudal levels, and the Mann-Whitney *U* test was used to analyze non-normally distributed data. The *χ*^2^ test and Fisher exact test were used to analyze categorical data. The rank-sum test was used to determine the difference of the fusion rate at C3–C7 absolute positions. Logistic regression analysis was used to identify risk factors for non-fusion. SPSS software version 23.0 (IBM Corporation, Armonk, NY, USA) was used to perform standard statistical analyses. A two-tailed *p* value < 0.05 was considered significant.

## Results

### Patient demographics

Eleven patients were excluded because three had tumors, two had tuberculosis infection, and six were lost to follow-up. In total, 118 patients and 236 fusion levels with at least 2-year follow-up were enrolled in this study, including 57 women and 61 men, with a mean age of 54.6 (range, 29–75) years. The mean duration of follow-up was 35.7 (range, 25–81) months. Detailed demographic data are summarized in Table 1.

**Table 1:** Demographic information

	Variable
**Sex (female/male)**	57/61
**Age (years)**	54.63 ± 10.94
**Follow-up time (month)**	35.7 ± 11.22
**BMI (kg/m**^**2**^**)**	23.89 ± 4.21
**Smoker (%)**	39 (33.06%)
**Operative level (%)**	
**C3/4, C4/5**	13 (11.02%)
**C4/5, C5/6**	58 (49.15%)
**C5/6, C6/7**	47 (39.83%)

*BMI* body mass index

Data are presented mean ± standard deviation or number (%)

### Fusion rate

**Fig. 2:**
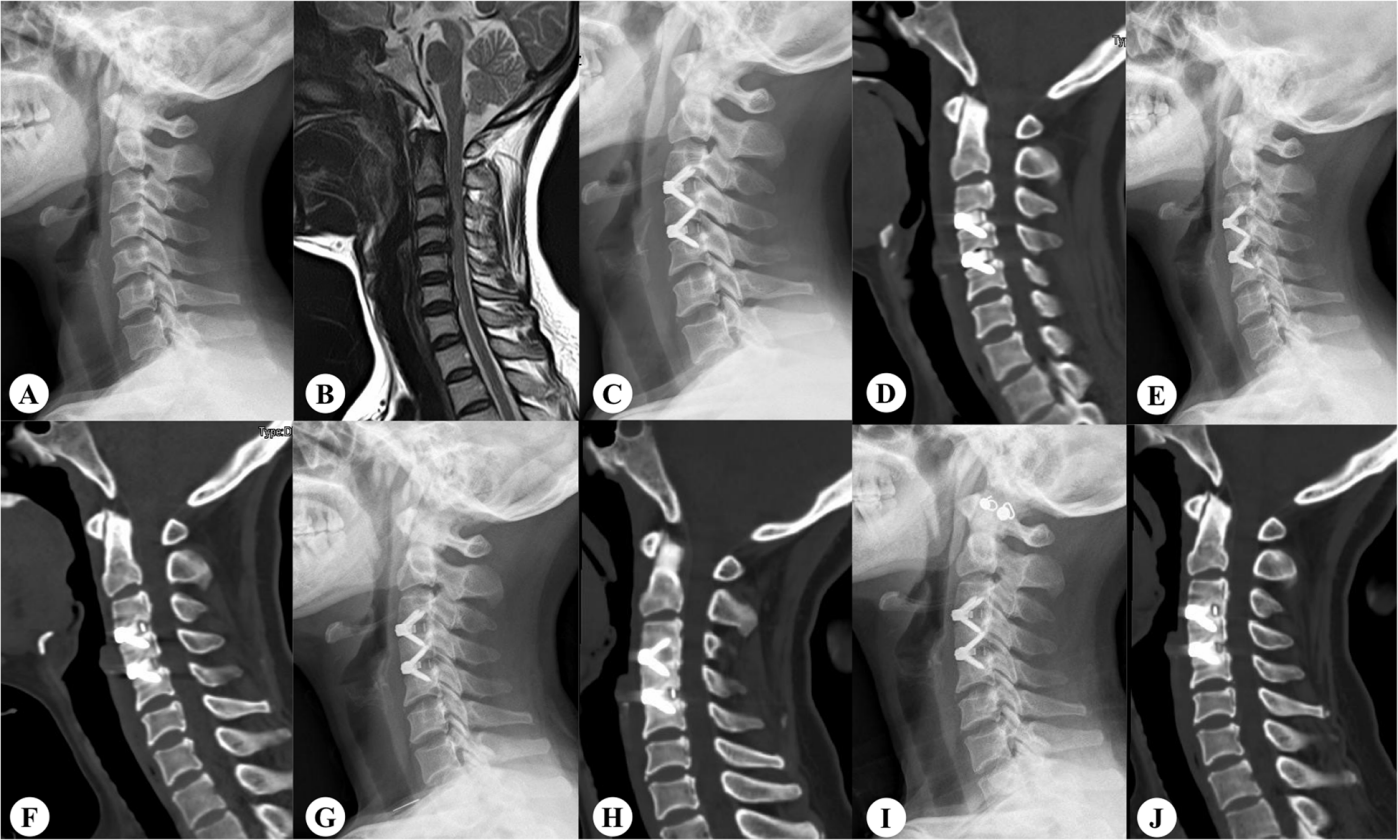
Radiographs of a 49-year-old woman. **A**, **B** Preoperative lateral radiograph showing degeneration and compression at the C3/4 and C4/5 levels. **C**, **D** Postoperative image showing two suitable prostheses placed at those levels. **E**, **F** Three-month postoperative lateral radiograph showing a growing bridge only at the cranial level. **G**, **H** Fusion of only the cranial level at 6 months postoperatively. **I**, **J** At 2 years postoperatively, fusion of only the cranial level is complete

Among the 118 patients, 13 (11.01%) underwent ACDF at the C3–C5 levels, 58 (49.15%) at the C4–C6 levels, and 47 (39.83%) at the C5–C7 levels. Overall, 106 (89.83%) patients achieved two-level fusion at the last follow-up. Moreover, three (2.54%) patients had only one-level fusion and nine (7.62%) had two-level non-fusion. A significant difference was found in the fusion rates between the cranial and caudal levels at 3 and 6 months postoperatively (Fig. 2). In all patients with fusion at the caudal levels, fusion at the cranial levels had already been achieved at 3 months postoperatively. Only seven (5.93%) patients had fusion at the caudal levels and non-fusion at the cranial levels at 6 months postoperatively. Interestingly, this difference disappeared at the 12-month and last follow-ups. Additionally, the non-fusion group had a larger preoperative segment slope than the fusion group. No significant difference was noted in the preoperative segment ROM and preoperative DH between the two groups (Table 2). Among the 236 levels, 13 (5.51%) were at the C3/4 level, 71 (30.08%) at the C4/5 level, 105 (44.49%) at the C5/6 level, and 47 (19.92%) at the C6/7 level. A total of 215 (91.1%) levels achieved fusion at the last follow-up. The fusion rate differed between the distribution of the operative segments at 3, 6, and 12 months (*p* < 0.05) (Table 3). The C6/7 level accounted for the caudal fusion rate compared with the C3/4 and C4/5 levels (Bonferroni-adjusted, *p* < 0.008) at 3 months postoperatively.

**Table 2:** Influential factors of the fusion rate between the fusion and non-fusion groups in 236 levels during the follow-up

		3 months			6 months			12 months			Last FU		
		Fusion	Non-fusion	***p***	Fusion	Non-fusion	***p***	Fusion	Non-fusion	***p***	Fusion	Non-fusion	***p***
**Relative position**	**Cranial levels**	31 (26.27%)	87 (73.72%)	0.0024	69 (58.47%)	49 (41.53%)	0.0191	102 (86.44%)	16 (13.56%)	0.4739	109 (92.37%)	9 (7.63%)	0.6484
	**Caudal levels**	12 (10.17%)	106 (89.83%)		50 (42.37%)	68 (57.63%)		97 (82.20%)	21 (17.80%)		106 (89.83%)	12 (10.17%)	
**PRE-segment slope (°)**		15.47 ± 4.787	18.42 ± 4.962	0.0005	16.69 ± 4.356	19.06 ± 5.406	0.0003	17.38 ± 4.704	20.67 ± 5.942	0.0002	17.57 ± 4.756	21.20 ± 6.702	0.0016
**PRE-ROM (°)**		6.604 ± 3.044	7.224 ± 3.841	0.3225	6.860 ± 3.327	7.366 ± 4.062	0.2957	7.056 ± 3.582	7.408 ± 4.381	0.5972	7.058 ± 3.679	7.658 ± 4.074	0.4802
**PRE-disk height (mm)**		4.71 ± 1.29	4.66 ± 1.09	0.7929	4.71 ± 1.17	4.62 ± 1.09	0.5395	4.70 ± 1.02	4.50 ± 1.00	0.3137	4.67 ± 1.15	4.68 ± 0.96	0.9753

*FU* follow-up, *PRE* preoperative, *ROM* range of motion

**Table 3:** Fusion rates and segment distributions

	3 months				6 months				12 months				Last FU			
	Fusion	Non-fusion	Fusion rate	***p***	Fusion	Non-fusion	Fusion rate	***p***	Fusion	Non-fusion	Fusion rate	***p***	Fusion	Non-fusion	Fusion rate	***p***
**C3/4**	5	8	38.46%	< 0.001	8	5	61.54%	0.044	12	1	92.31%	0.028	13	0	100%	0.148
**C4/5**	18	53	25.35%		40	31	56.34%		59	12	83.10%		66	5	92.96%	
**C5/6**	18	87	17.14%		53	52	50.48%		89	16	84.76%		95	10	90.48%	
**C6/7**	2	45	4.26%		18	29	38.30%		39	8	82.98%		41	6	87.23%	

*FU* follow-up

Logistic regression analysis was performed to confirm that both the relative position (3 months, *B* = 0.987, odds ratio [OR] = 2.684, 95% confidence interval [CI] 1.075–6.698, *p* < 0.05; 6 months, *B* = 0.859, OR = 2.362, 95% CI 1.192–4.687) and preoperative segments slope (3 months, *B* = −0.100, OR = 0.905, 95% CI 0.840–0.976, *p* < 0.05; 6 months, B = −0.101, OR = 0.920, 95% CI 0.868–0.974, *p* < 0.001) were predictive factors of fusion success. Segment distribution was not related with fusion success (Table 4).

**Table 4:** Multivariate logistic regression analysis of the influential factors of the fusion rate

	3 months				6 months			
	***ß***	OR	95% CI	***p***	***ß***	OR	95% CI	***p***
**Relative position**	0.987	2.684	1.075–6.698	0.034	0.859	2.362	1.192–4.687	0.014
**Preoperative segment slope**	−0.1	0.905	0.840–0.976	0.009	−0.101	0.904	0.852–0.959	0.014
**C3/4**	1.073	2.924	0.359–23.788	0.316	−0.520	0.595	0.132–2.688	0.500
**C4/5**	0.951	2.588	0.458–14.643	0.282	−0.398	0.671	0.255–1.770	0.420
**C5/6**	0.860	2.362	0.466–11.971	0.299	0.041	1.042	0.476–2.284	0.918
**C6/7**	-	1	-	-	-	1	-	-

*CI* confidence interval, *OR* odds ratio

### Complications

Subsidence was detected in 22 (9.32%) levels at the last follow-up. Radiographic ASD was detected in 55 (23.31%) levels at the last follow-up. However, no patient required adjacent-segment secondary surgery during the follow-up. In addition, no significant difference concerning ASD or subsidence was observed between the cranial and caudal levels (Table 5). No screw loosening, displacement, vertebral body osteolysis, or vertebral fracture was reported in all patients. No patient required implant-related secondary surgery.

**Table 5:** Comparison of complications between the cranial and caudal levels at the last follow-up

	**Cranial levels**	**Caudal levels**	***p***
**Subsidence (rate)**	10 (8.47%)	12 (10.17%)	0.824
	**Cranial adjacent segment**	**Caudal adjacent segment**	
**ASD (rate)**	32 (27.12%)	23 (19.49%)	0.218

*ASD* adjacent segment degeneration

## Discussion

Fusion is an important criterion to determine the success of ACDF. The criteria for judging whether fusion has been achieved have not been established yet [21], which affects the observed values of the fusion rate. After reviewing the literature, we believe that the criterion used in our study is accurate. In recent studies, the fusion rate of two-level ACDF ranged from 91.4 to 100% [22–25], with at least 1 year of follow-up. However, the regularity of early postoperative fusion (3–6 months) is the key to accelerated rehabilitation. Unfortunately, there were few related studies on this topic. The results of the present study reveal the early fusion rule after two-level ACDF, which may provide a reference for adjusting the bone grafting strategy. Some bone chips or bone dust are often produced during decompression. These bone tissues can be transplanted back to the intervertebral space as an autograft to promote fusion. However, in two-level ACDF, these bones are often enough to fill only one intervertebral space. Thus, this study provides a reference for which intervertebral space should be filled.

The fusion rate of the cranial and caudal levels was different in two-level ACDF. Wang et al. [17] found that all pseudarthrosis in multi-level ACDF occurred at the caudal level with an average follow-up of 3.2 years. Nichols et al. [18] also found that the fusion rate was lower at 24 months postoperatively in multi-level ACDF when using the plate with a cage system. McClure et al. [26] reported that only 44% and 42% of multi-level ACDFs achieved fusion at the C6–7 and C7–T1 levels, which was significantly lower than that for the C3–6 level. However, no study has compared the fusion rate between the cranial and caudal levels with the zero-profile device in ACDF. Because of the high mobility of the cervical vertebra and stiffness of the thoracic vertebra, there is tremendous biomechanical pressure at the cervicothoracic junction, and this area is prone to degradation [27, 28]. In two-level ACDF, the caudal segment is closer to the cervicothoracic junction, so the potential activity of the caudal segment was higher, which may be the reason for its caudal fusion rate. In addition, zero-profile fixation is less robust than plate fixation, which may lead to greater potential activity, resulting in a higher incidence of non-fusion. Through the preliminary analysis, we found that the fusion rate was related to the absolute and relative positions of the segments. To confirm the effect of these positions on the fusion rate and exclude confounding factors, we included these variables and the segment slope into the multivariate logistic regression analysis and found that the caudal level was an independent risk factor of non-fusion. A biomechanical study [29] reported that C6/7 is the first segment and the most active segment of the cervical spine to move during flexion and extension, and it moves much more than other segments, which means that it receives more force during cervical movement and reduces the fusion rate. Interestingly, no difference was noted in the rates of subsidence and incidence of ASD between the cranial and caudal segments.

The fusion rate may be related to the preoperative segment slope in two-level ACDF. The larger segment slope tends to mean larger shear stress. Other studies [16, 30, 31] have found that the preoperative T1 slope is a risk factor for non-fusion or pseudarthrosis. Although the T1 slope is a conventional base for describing cervical sagittal position force lines, it was not measurable in nearly 70% of the patients [32, 33]. Certain differences were also noted in the slope of each segment, so the T1 slope cannot directly reflect the slope of the index level. Therefore, this study directly measured the slope of the two index levels and determined its relationship with the fusion rate.

This study has some limitations. First, this was a retrospective study, and there may be inherent potential bias. Second, the results of the 2-year follow-up might have underestimated the incidence of ASD. Thus, long-term follow-up studies are needed to confirm the incidence of ASD. Third, this study did not describe other possible risk factors for non-fusion. In this study, age, sex, smoking, osteoporosis, T1 slope, diabetes, and other confounding factors were well controlled by matching and comparing the fusion rates between the cranial and caudal segments. Thus, not all risk factors of non-fusion were elaborated and focused on in this study. Despite these limitations, to the best of our knowledge, this is the first study to focus on the fusion rate and order between the cranial and caudal levels in two-level ACDF in a large sample of patients. Moreover, this study provides us with more ideas on achieving early fusion.

## Conclusions

The caudal level had a slower fusion process than the cranial level. A higher preoperative segment slope was a risk factor of fusion. However, the subsidence and ASD rate were comparable between the caudal and cranial levels in two-level ACDF.

## Data Availability

The datasets in this study are available from the corresponding author on reasonable request.

## Abbreviations

ACDF: Anterior cervical discectomy and fusion
ASD: Adjacent segment degeneration
BMI: Body mass index
cDDD: Cervical disk degenerative disease
CI: Confidence interval
CT: Computed tomography
DH: Disk height
FU: Follow-up
MRI: Magnetic resonance imaging
OR: Odds ratio
ROM: Range of motion
